# MACF1 promotes preosteoblast migration by mediating focal adhesion turnover through EB1

**DOI:** 10.1242/bio.048173

**Published:** 2020-03-24

**Authors:** Peihong Su, Chong Yin, Dijie Li, Chaofei Yang, Xue Wang, Jiawei Pei, Ye Tian, Airong Qian

**Affiliations:** 1 Laboratory for Bone Metabolism, Key Laboratory for Space Biosciences and Biotechnology, School of Life Sciences, Northwestern Polytechnical University, Xi'an 710072, China; 2 Research Center for Special Medicine and Health Systems Engineering, School of Life Sciences, Northwestern Polytechnical University, Xi'an 710072, China; 3 NPU-UAB Joint Laboratory for Bone Metabolism, School of Life Sciences, Northwestern Polytechnical University, Xi'an 710072, China

**Keywords:** Bone, Cell migration, FA turnover, Preosteoblast

## Abstract

Microtubule actin crosslinking factor 1 (MACF1) is a widely expressed cytoskeletal linker and plays an essential role in various cells’ functions by mediating cytoskeleton organization and dynamics. However, the role of MACF1 on preosteoblast migration is not clear. Here, by using MACF1 knockdown and overexpressed MC3T3-E1 cells, we found MACF1 positively regulated preosteoblast migration induced by cell polarization. Furthermore, immunofluorescent staining showed that MACF1 increased end-binding protein (EB1) distribution on microtubule (MT), and decreased EB1 distribution on focal adhesion (FA) complex. Moreover, upregulation of MACF1 activated Src level and enhanced the colocalization of EB1 with activated Src. In addition, MACF1 diminished colocalization of EB1 with adenomatous polyposis coli (APC), which induced EB1 release from FA and promoted FA turnover. These results indicated an important role and mechanism of MACF1 in regulating preosteoblast migration through promoting FA turnover by mediating EB1 colocalization with Src and APC, which inferred that MACF1 might be a potential target for preventing and treating bone disorders.

## Abstract (precis)

**Summary:** This work illustrated MACF1 function and mechanisms in regulating preosteoblast migration, which provided a possibility that MACF1 could be considered as a potential target for bone disease therapy.

## INTRODUCTION

Preosteoblast or their precursors can migrate into bone resorption cavities and attach to the bottom of those cavities. These are critical events in maintaining bone mass (Dirckx et al., 2013; Shirakawa et al., 2014). Improving preosteoblast/precursor migration is a potential strategy for bone disease treatments, such as for osteoporosis and bone fractures (Su et al., 2018).

Polarization is the critical event for cell migration. During polarization and directed motility in wound healing, Golgi become positioned towards the wound edge. The position of Golgi body relative to the nucleus is considered to be a sensitive indicator for cell polarization. In addition, the ratio of length/width is another indicator for cell polarization (MeganE. Brasch et al., 2019). The reduction of cell polarization prevents the movement of microtubules (MT) plus end-tracking proteins (+TIPs) along MT bundles. End-binding protein 1 (EB1), as a member of +TIPs, binds the plus ends of growing MT and regulates MT stability and dynamics through recruiting other +TIPs to MT plus ends (Kodama et al., 2003). Moreover, interactions between EB1 and other +TIPs, such as adenomatous polyposis coli (APC) and CLASPs, are important for MT dynamics during cell migration (Mimori-Kiyosue et al., 2005). Additionally, EB1 is phosphorylated by activated proto-oncogenic protein Src (p[Y418] Src) that localizes strongly to FAs. This process also plays an essential role in cell migration (Webb et al., 2004; Zhang et al., 2004).

MACF1 (microtubule actin crosslinking factor 1) is a member of the spectraplakins superfamily, which can bind to both F-actin and MT (Karakesisoglou et al., 2000; Leung et al., 1999; Sun et al., 2001). As a ∼600 kDa cytoskeletal linker, MACF1 has been reported to enhance epidermis and vertebral cortex neuron migration and polarization, via controlling MT organization and MT-FA (focal adhesion) dynamics (Ka et al., 2014; Wu et al., 2011). In addition, MACF1 deficiency changed the position of the nucleus relative to the Golgi body, a responsive indicator for cell polarization in keratinocytes. Wu et al. reported that MACF1 deficiency reduced keratinocyte migration and inhibited FA dynamics (Wu et al., 2008). Moreover, MACF1 was highly expressed in preosteoblasts, and the co-localization of MACF1 and the cytoskeleton was affected in preosteoblasts when MACF1 expression levels were altered by environmental stimuli (Qian et al., 2009). Furthermore, MACF1 knockdown induced cytoskeleton redistribution in MC3T3-E1 cells (Hu et al., 2015). However, the regulation by MACF1 of preosteoblast migration and its molecular mechanism are still unclear.

In the present study, to further reveal the function of MACF1 in preosteoblast migration, MACF1 knockdown and MACF1 overexpression MC3T3-E1 cell models were established and adopted to evaluate cell migration and polarization. Then the distribution of EB1 and colocalization of EB1 with p[Y418] Src and APC was determined to uncover the mechanism. These results showed a novel role of MACF1 on mediating preosteoblast migration and offered a new insight into both MACF1 and preosteoblast function.

## RESULTS

### MACF1 enhanced preosteoblast cell migration *in vitro*

**Fig. 1.**
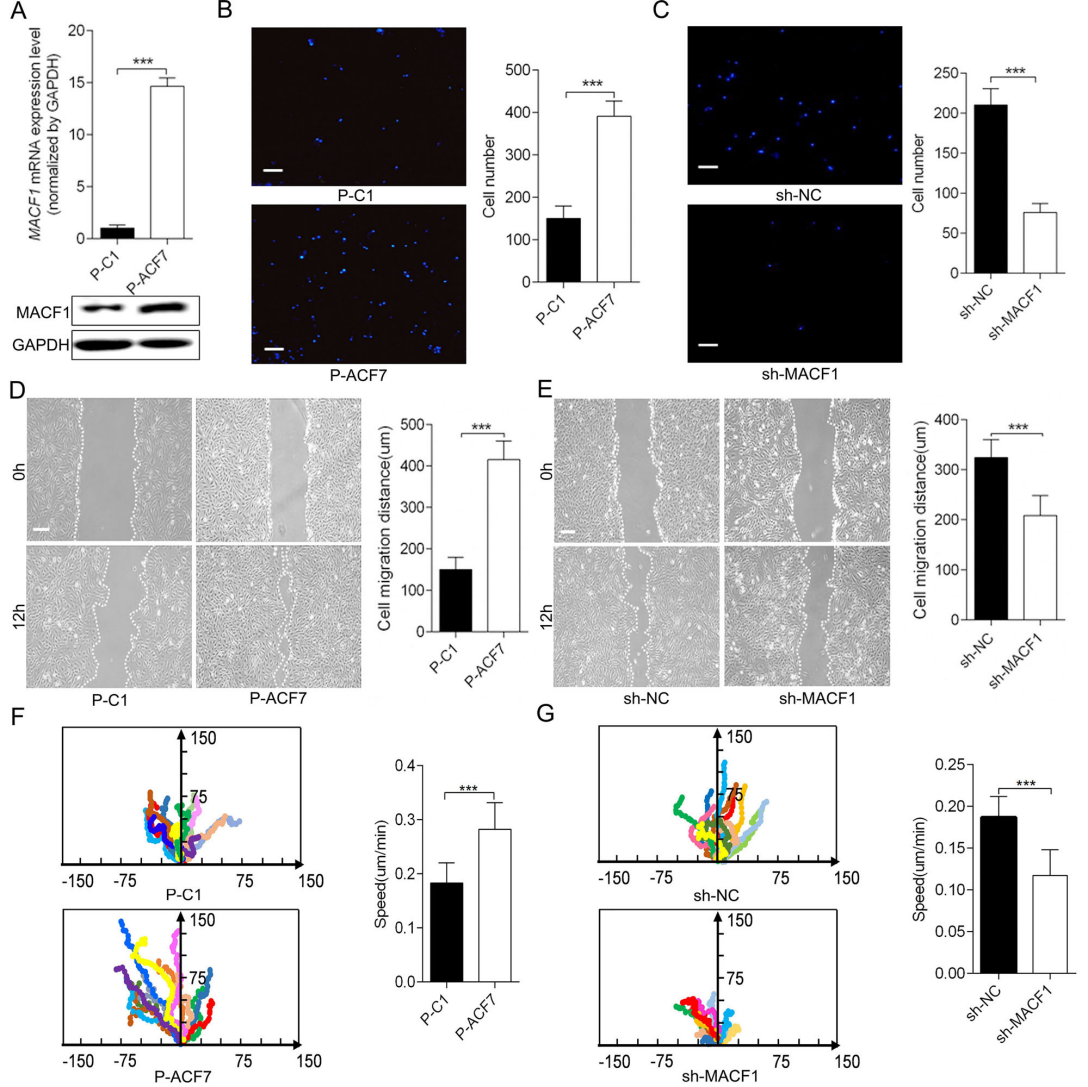
**MACF1 enhances MC3T3-E1 preosteoblast migration *in vitro.*** (A) Establishment of MACF1 overexpressed MC3T3-E1 cell. (B,C) The number of cells that had migrated to the lower chamber was visualized with DAPI and quantitatively analyzed after 12 h incubation (*n*=3). (D,E) Migration distance of the cell was measured and quantitatively analyzed. (F,G) Migration trajectory of individual cells was monitored by time-lapse videomicroscopy and cell migration speed was quantitatively analyzed (*n*=20). (B,D,F) MACF1 overexpression MC3T3-E1 cell; (C,E,G) MACF1-knockdown MC3T3-E1 cell. Mean±s.d., ***P<0.001. Scale bars: for B and C: 100 μm for D and E: 200 μm.

Monoclonal MACF1 overexpressed preosteoblast (P-ACF7) and control cell (P-C1) models were established and MACF1 expression was confirmed at both the RNA and protein level (Fig. 1A; Fig. S1). Cell migration was detected using a transwell chamber assay and a wound healing assay. As shown in Fig. 1B, MACF1 overexpression increased the number of migratory MC3T3-E1 cells by 50.7±7.6% (Fig. 1B, *P*<0.001), and increased MC3T3-E1 migration distance by 62.5±0.9% compared to P-C1 (Fig. 1D, *P*<0.001). Conversely, for MACF1 knockdown MC3T3-E1 (sh-MACF1), migratory cell numbers decreased by 64.3±4% (Fig. 1C), and cell migration distance was reduced by 37.5±0.9% (Fig. 1E, *P*<0.001), compared to the negative control (sh-NC).

We used videomicroscopy to monitor the speed and directed migration of individual MC3T3-E1 cells. The representative cells were highlighted to show the movement traces (Fig. 1F,G). As the results demonstrated, over-expression of MACF1 made the trajectory of cells more directional and straighter, while knocking down MACF1 led to random and curved movement of cells. The speed of P-ACF7 cells was also significantly faster than P-C1 cells, but sh-MACF1 cells moved much more slowly than sh-NC cells. Quantification of these movement speeds revealed that MACF1 overexpression increased preosteoblast migration speed by 47.3% (Fig. 1F, *P*<0.001) and MACF1 knockdown decreased migration speed by 33.3% (Fig. 1G, *P*<0.001), as compared to their control cells. These results suggested that MACF1 might promote osteoblast migration *in vitro*.

### MACF1 promoted preosteoblast migration *in vivo*

**Fig. 2.**
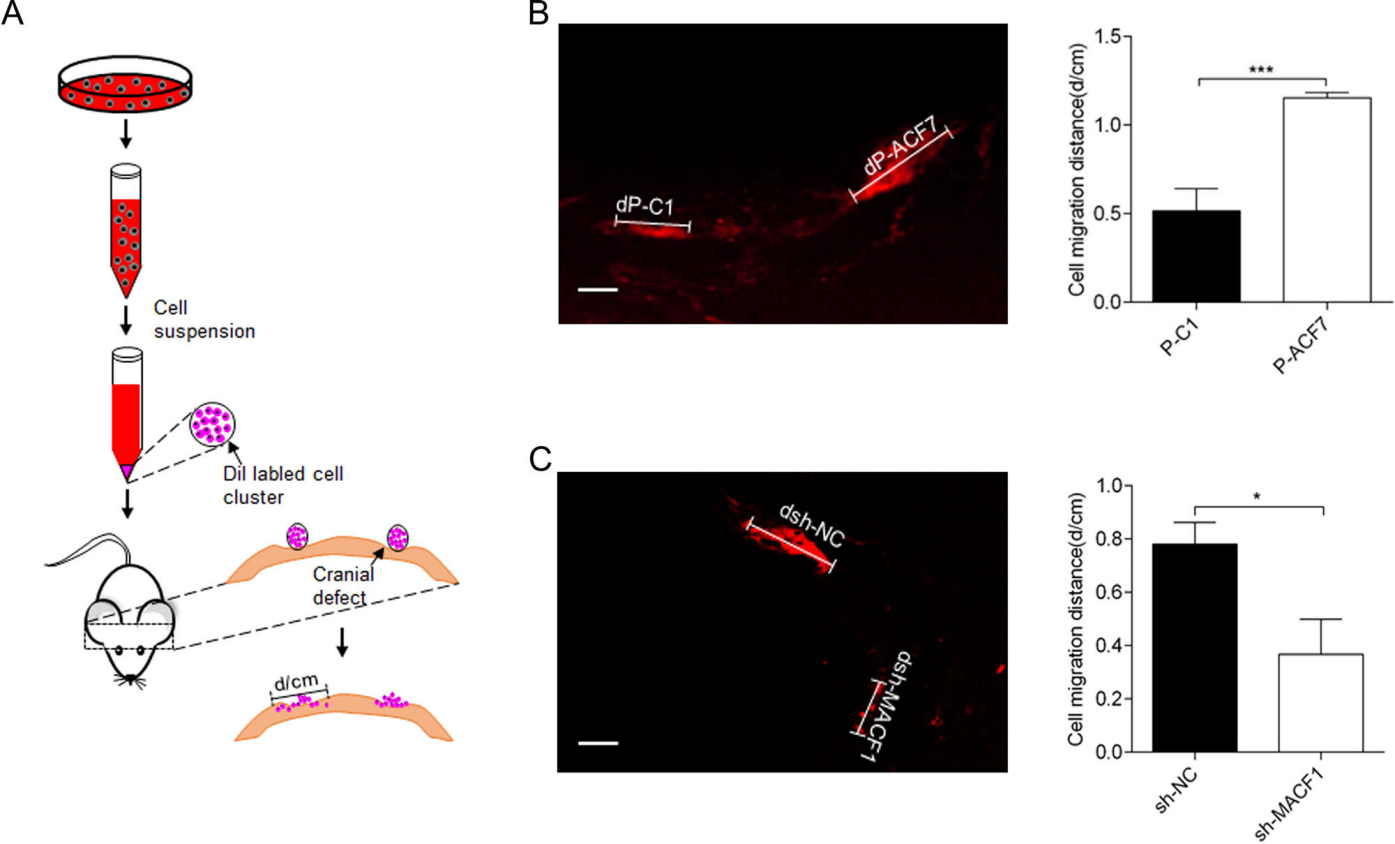
**MACF1 promotes MC3T3-E1 preosteoblast migration *in vivo.*** (A) Schematic diagram of cell implantation method. (B,C) Migration distance was measured 2 weeks after cell implantation, (B) MACF1 overexpression MC3T3-E1 cell (dP-ACF7 compared to control dP-C1) (*n*=8), (C) MACF1-knockdown MC3T3-E1 cell (dsh-MACF1 compared to control dsh-NC) (*n*=8). Representative pictures and quantification of migration distance are shown. Mean±s.d. Scale bars: 200 μm, *P<0.05, ***P<0.001.

To further confirm the function of MACF1 in preosteoblast cell migration *in vivo*, Dil^+^ MC3T3-E1 cells were implanted into mouse calvarial defects (Fig. 2A). After 2 weeks of implantation, the distance of Dil^+^ cell expansion in the calvaria was examined. MACF1 overexpression increased the Dil^+^ cell expansion distance (dP-ACF7) in calvarial defects by 42±5%, compared to control (dP-C1) (Fig. 2B). In contrast, the distance of Dil^+^ sh-MACF1 cells (dsh-MACF1) was decreased by 50±1.9%, compared to their control (dsh-NC) (Fig. 2C). These results were identical with the *in vitro* findings.

### MACF1 enhanced preosteoblast polarization during cell migration

**Fig. 3.**
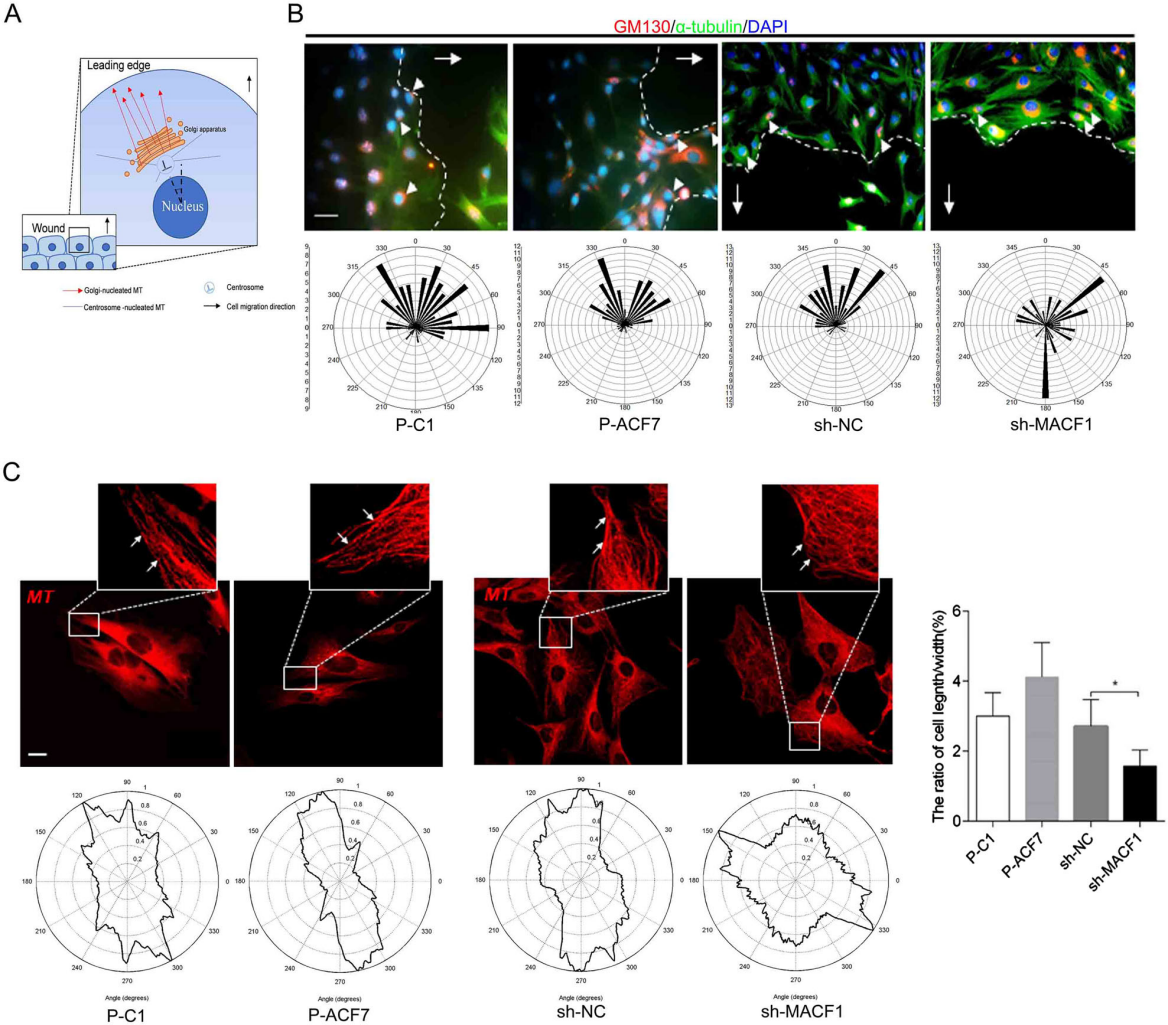
**MACF1 enhances MC3T3-E1 cell polarization.** (A) Schematic diagram of microtubule and Golgi apparatus in migrating cell at the edge of wound. (B) The angle of the Golgi complex relative to the long axis was detected using immunostaining. The white arrows refer to cell migration direction, the white arrowheads indicate Golgi position. Representative pictures were shown (upper panels); wind rose plots of the angles were shown (lower panels). Scale bar: 100 μm, *n*=80. (C) Quantification of length/width ratio of cell at the outer edge of wound. Mean±s.d., *n*=40, *P<0.05.

We further investigated the effect of MACF1 on MC3T3-E1 cell polarization during migration. As shown in Fig. 3A, orientation of the long axis that was perpendicular to the wound direction was determined. Then the angle of the Golgi complex relative to the long axis was measured. Wind rose plots of the angles showed that the angles of most P-C1 cells fell between a 270–90° range and P-ACF7 cells fell between 300–60°. Moreover, the angles in sh-MACF1 cells appeared to be random and irregular, which was significantly different from the angles in sh-NC cells (300–45°) (Fig. 3B). Further results demonstrated that MACF1 overexpression mildly increased the length/width ratio. However, MACF1 knockdown significantly decreased the length/width ratio (Fig. 3C). All these results illustrated that MACF1 enhanced preosteoblast cell polarization, which further promoted preosteoblast migration.

### MACF1 influenced EB1 distribution on MT bundle and FA complex

**Fig. 4.**
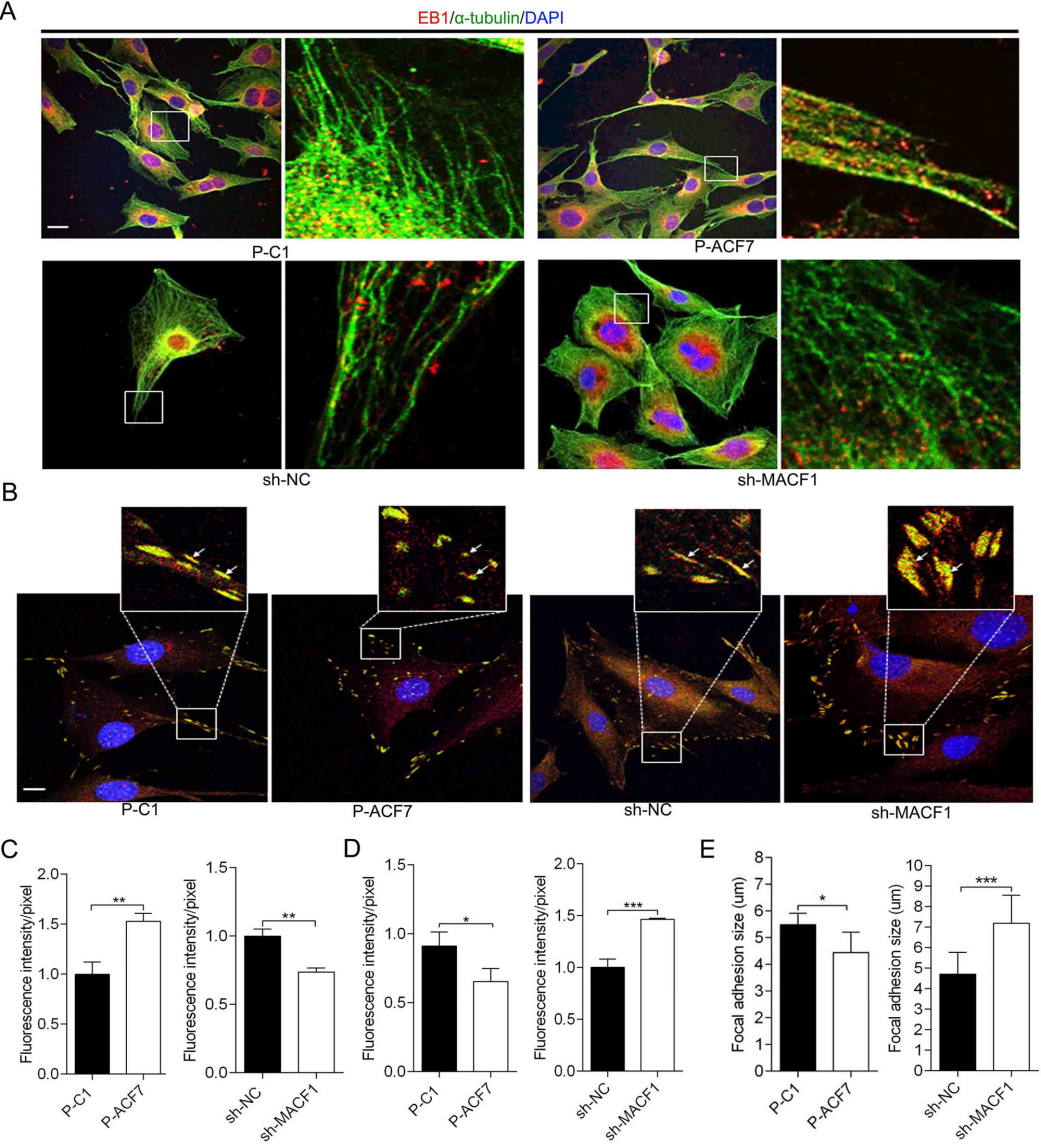
**MACF1 influences EB1 localization on MT bundle and FA (focal adhesion).** EB1 distribution on MT bundle (A) and FA complex (B) in MC3T3-E1 cell are visualized by immunofluorescence staining. The white arrows indicate the distribution of EB1 on FA complex. Quantification analysis of the distribution of EB1 on MT bundle (C) and FA complex (D) in MC3T3-E1 cells. (E) Quantification analysis of FA size in MC3T3-E1 cell (*n*=50, mean±s.d.). Representative pictures are shown. Scale bars: 50 μm. *P<0.05, **P<0.01, ***P<0.001.

As mentioned above, the reduction of cell polarization is associated with +TIPs movement along the MT bundle. To gain further insight into the role of MACF1 in MC3T3-E1 cell polarization, EB1 distribution on the MT bundle was initially determined. The results showed that EB1 extended in a straight direction towards the MT growth orientation both in P-C1 and P-ACF7 cells, while in P-ACF7 cells more EB1 localized at the MT bundle compared to P-C1 cells. However, in sh-MACF1 cells most of EB1 was localized near the cell nucleus and little EB1 was visualized at the MT bundle, compared to sh-NC cells (Fig. 4A). Quantitative analysis of EB1 distribution on MT bundles showed that MACF1 overexpression increased EB1 distribution on MT bundles by 50%, compared to control cells, and MACF1 knockdown reduced EB1 distribution by 30%, compared to control cells (Fig. 4B). These results suggested that MACF1 enhanced EB1 movement along the MT bundle to MT plus end in preosteoblasts.

EB1 distribution on FA was also detected. The results showed that less EB1 localized on FA complex and colocalized with vinculin in P-ACF7 cells, compared to P-C1 cells. In addition, in sh-MACF1 cells, more EB1 localized on FA and colocalized with vinculin, compared to sh-NC cells (Fig. 4C). Quantitative analysis of EB1 distribution on FA complex revealed that MACF1 overexpression decreased distribution of EB1 on FA complex by 12.5% compared to control cells, and MACF1 knockdown increased by 40%, compared to control cells (Fig. 4D).

Moreover, we found FA shape and size were changed by different MACF1 expression levels. In P-ACF7 cells, FA shape was punctiform and irregular compared to P-C1 cells, while in sh-MACF1 cells, FA was wide, long and triangular compared to the slender shape in sh-NC cells. Quantitative analysis of FA size showed that average FA size was smaller in P-ACF7 cells (4.443±0.2869 μm), compared to P-C1 cells (5.480±0.1960 μm, *P*<0.05). While the average size of FA was significantly larger in sh-MACF1 cells (7.192±0.3799 μm), compared to sh-NC cells (4.713±0.2731 μm, *P*<0.001) (Fig. 4E). EB1 distribution pattern and FA size were significantly changed in MC3T3-E1 cells with different MACF1 levels, which implied that MACF1 might regulate FA turnover during cell migration. Conclusively, these results revealed that MACF1 enhanced EB1 movement along the MT bundle and FA turnover in preosteoblasts.

### MACF1 diminished interaction of EB1 with APC by influencing Src activation

**Fig. 5.**
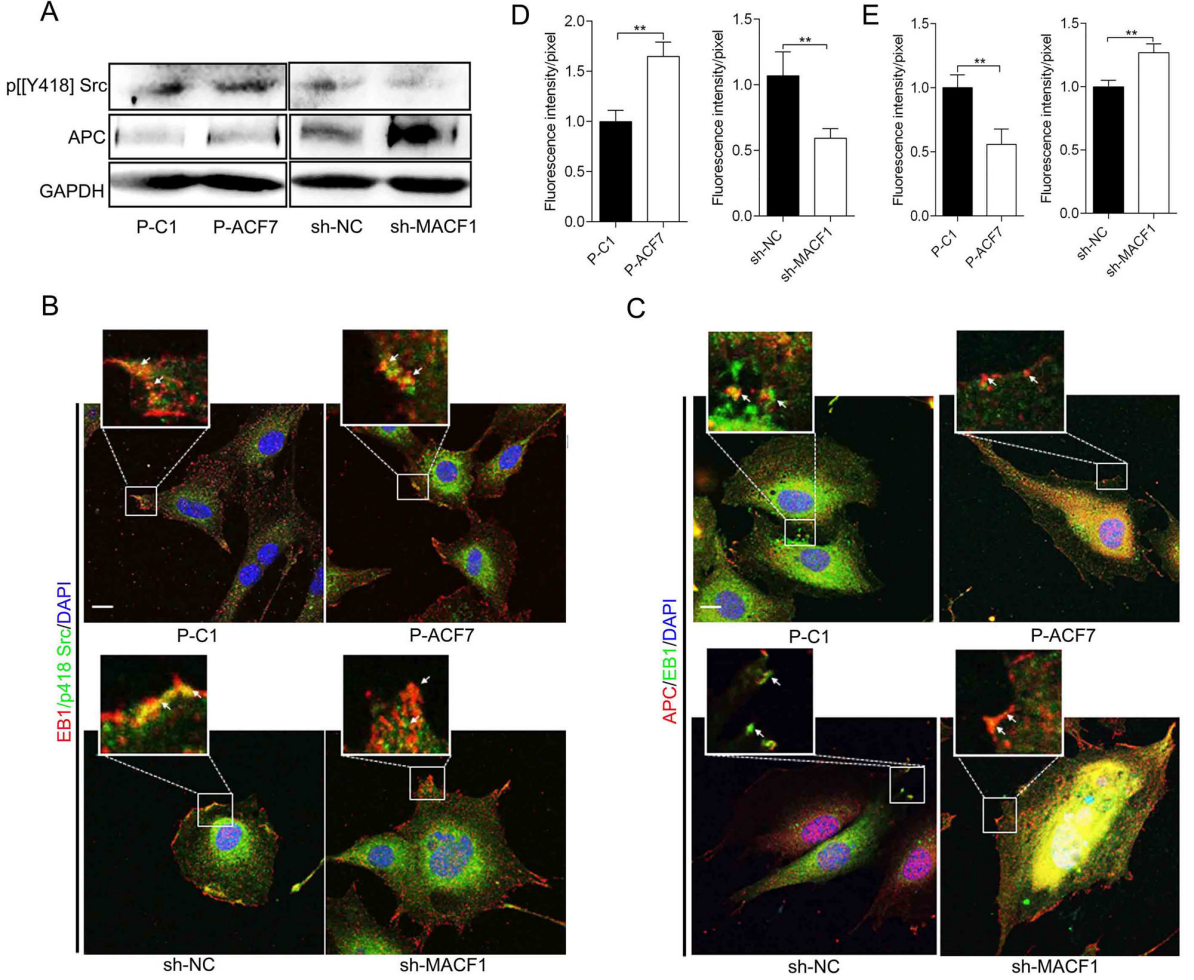
**MACF1 diminished interaction of EB1 with APC by influencing Src activation.** (A) p[Y418] Src and APC levels in MC3T3-E1 cells are detected by western blot. Colocalization of EB1 with p[Y418] Src (B) and APC (C) on FA complex was detected by immunofluorescence staining. The white arrows indicate colocalization of EB1 with P[Y418] Src or APC. Scale bars: 50 μm. Quantification analysis of colocalization of EB1 with p[Y418] Src (D) and APC (E) was shown. Mean±s.d., **P<0.01.

Phosphorylated Src (p[Y418] Src) indicated the activation status of Src. We detected p[Y418] Src levels in MC3T3-E1 cells due to its essential role in regulating the interaction of EB1 with other +TIPs and FA turnover. The results showed that p[Y418] Src levels were not altered by MACF1 overexpression, but significantly downregulated in sh-MACF1 cells compared to sh-NC cells (Fig. 5A). Immunofluorescent staining indicated that there was a strong colocalization of EB1 with p[Y418] Src in P-ACF7 cells, compared to P-C1 cells. However, in sh-MACF1 cells, the colocalization was weak and most EB1 was dissociated from p[Y418] Src, compared to in sh-NC (Fig. 5B). Quantitative analysis demonstrated that MACF1 overexpression reinforced colocalization of EB1 with p[Y418] Src by 70% compared to control cells, and MACF1 knockdown decreased colocalization by 36.4% compared to control cells (Fig. 5D). These data indicated that MACF1 increased p[Y418] Src levels and promoted colocalization of EB1 with p[Y418] Src. P[Y247] EB1, phosphorylated at Y247 by activated Src, tended to move along the MT bundle, instead of being anchored at the FA complex. The result may denote a high EB1 phosphorylation level in preosteoblasts.

We also noticed obviously upregulated APC levels in sh-MACF1 cells compared to sh-NC cells. Immunofluorescent staining revealed that the colocalization of EB1 with APC decreased in P-ACF7 cells compared to P-C1 cells. However, in sh-MACF1 cells, the colocalization was increased significantly and most of the EB1 was covered by APC compared to sh-NC cells (Fig. 5C). Quantitative analysis showed that MACF1 overexpression decreased colocalization of EB1 with APC by 40% and MACF1 knockdown increased colocalization by 20% compared to their control (Fig. 5E).

These results illustrated that MACF1 weakened the interaction of EB1 with APC and MACF1 knockdown increased APC level, which induced EB1 was gripped by APC at FA complex, instead of moving along MT bundle and FA was stabled in preosteoblast.

## DISCUSSION

Preosteoblasts are the main functional cells in bone formation. Dysfunction of preosteoblasts will lead to bone disorders. Currently, most studies mainly focus on preosteoblast/osteoblast differentiation and proliferation to clarify pathogenesis of bone disorder. However, preosteoblast cell migration is indispensable for bone formation. Abnormal preosteoblast cell migration will induce bone disorder as well, such osteoporosis and delay of fracture healing.

As a cytoskeletal protein, MACF1 is widely expressed in different tissues and correlates with a number of physiological and pathological processes (Hu et al., 2016; Suozzi et al., 2012). It has been reported that MACF1 regulates cell migration through mediating MT organization and dynamics in keratinocytes and neurons (Ka et al., 2014; Wu et al., 2011). Our previous study showed that MACF1 is highly expressed in preosteoblasts indicating MACF1 may have an important function in preosteoblasts and bone diseases. In this study, we first found that MACF1 promoted MC3T3-E1 cell migration *in vitro* and *in vivo*, which implies that MACF1 is a positive regulator for bone formation.

The position of the Golgi body relative to the position of the nucleus is the indicator of cell polarization and directed motility. In this study, we found MACF1 positively regulated MC3T3-E1 cell polarization, which is consistent with the phenomenon observed in keratinocyte and neuron cells (Wu et al., 2011). This means MACF1, as a broadly expressed protein, has a common function in different tissues and cells. Furthermore, Golgi polarization is associated with derived vesicle transportation during cell migration. While MACF1 has been reported as a member of Golgi derived vesicle transporter (Lin et al., 2005). Therefore, MACF1 may influence MC3T3-E1 cell polarization and migration by mediating vesicle transportation.

It is reported that, during cell migration, the distribution and interaction of +TIPs maintains MT stability and dynamics (Wu et al., 2011). Activated Src phosphorylates EB1 at Y247 and diminishes the interaction of EB1 with APC. Phosphorylated EB1 will be released from the FA complex and move along the MT bundle to stimulate cell migration. However, the function and associated mechanisms of MACF1 in regulating the activation of Src is still unclear. We revealed that MACF1 increased EB1 distribution on the MT bundle and decreased distribution on the FA complex in MC3T3-E1 cells. Moreover, we clarified that MACF1 upregulated p[Y418] Src levels and enhanced colocalization of EB1 and p[Y418] Src, which implied a high level phosphorylated EB1 at FA complex in MC3T3-E1 cells. Additionally, we discovered that MACF1 decreased APC levels and diminished colocalization of APC with EB1, which further enhanced the release of EB1 from the FA complex and promoted FA turnover. We also found MACF1 negatively affected FA size in MC3T3-E1 cells, which is consistent with Wu et al.’s work (Wu et al., 2008). Our results illustrate that MACF1 activates Src and then enhances EB1 release from the FA complex. The size changes in MC3T3-E1 cells with different MACF1 levels might result from the EB1 anchor on the FA complex.

MACF1 participates in the Wnt/β-catenin pathway and regulates preosteoblast function. MACF1 knockdown inhibits the Wnt/β-catenin pathway during preosteoblast differentiation (Hu et al., 2018). In this work, we found APC, as a component of the Wnt/β-catenin pathway was upregulated in MACF1-knockdown MC3T3-E1 cells, which may be associated with the inhibition of ubiquitination degradation of APC in Wnt/β-catenin. The data suggests MACF1 could regulate preosteoblast migration through the Wnt/β-catenin pathway, which deserves further study.

**Fig. 6.**
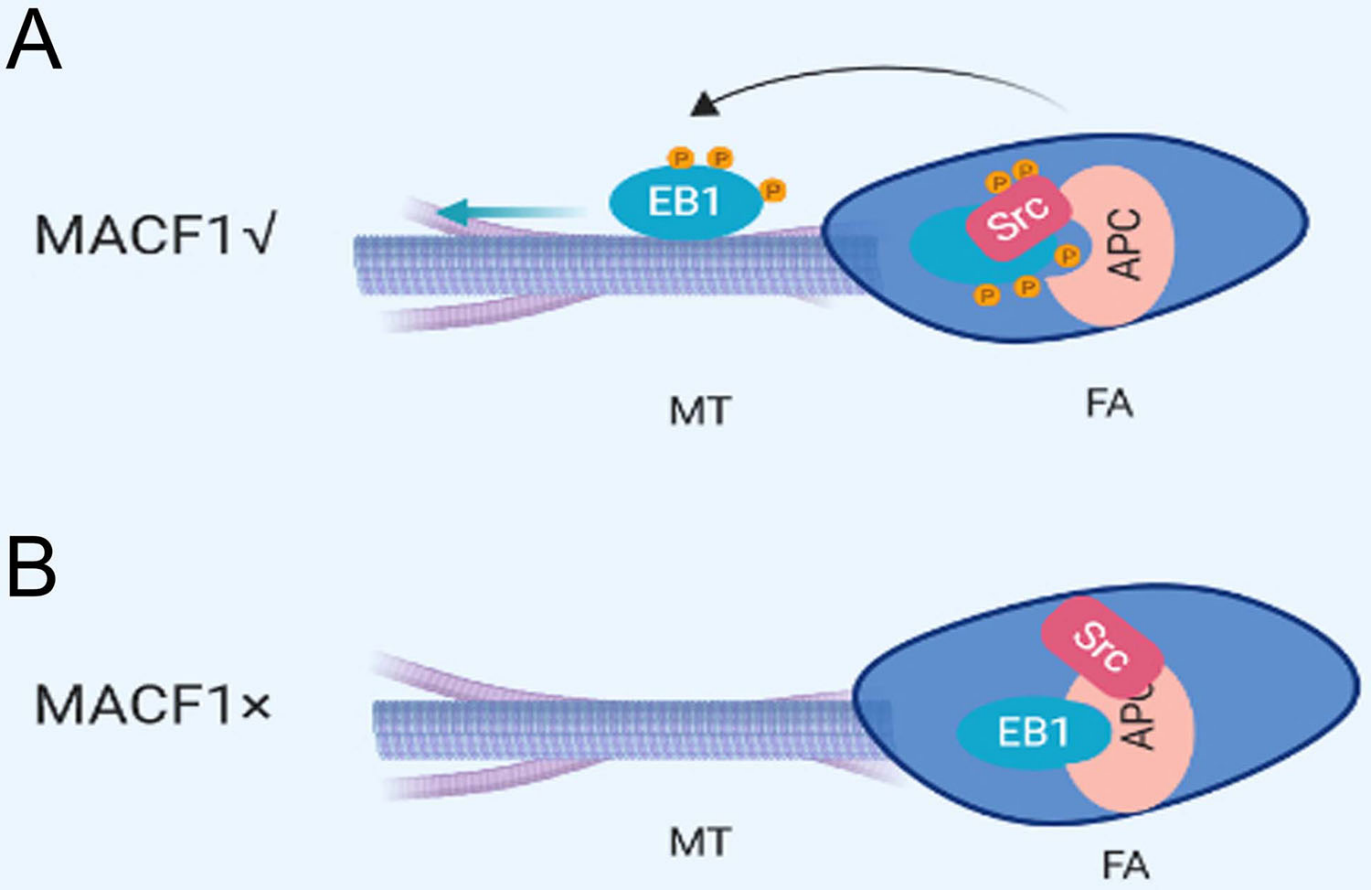
**The model for MACF1 regulating** **MT-FA network.** (A) With MACF1 expression. Activated Src phosphorylate EB1, which diminishes the binding of EB1 with APC and leads to EB1 release from the FA complex. The black curved arrow represents release; the straight blue arrow represents the direction of movement. (B) Without MACF1 expression. Src is not activated and EB1 is gripped at the FA complex by APC.

In conclusion, the current study revealed that MACF1 positively regulated preosteoblast migration. This work also demonstrated that the promotional effect of MACF1 on preosteoblast migration might be due to increased cell polarization. Furthermore, this study indicated the mechanism of MACF1 regulated preosteoblast migration through mediating FA turnover, through influencing the interaction of EB1 with APC via changing Src activation (Fig. 6).

Taken together, this study discovered a novel role of MACF1 in regulating preosteoblast functions and provided new insights and potential targets for preventing and treating bone disorders.

## MATERIALS AND METHODS

### Plasmids and cell cultures

MACF1 overexpression plasmid pEGFP-C1A-ACF7 was generously provided by Dr Xiaoyang Wu (The University of Chicago, Chicago, IL, USA). Control plasmid pEGFP-C1A was purchased from GeneChem (Shanghai, China).

Murine preosteoblast cell line MC3T3-E1 was generously provided by Dr Hong Zhou (The University of Sydney, Sydney, Australia). MC3T3-E1 cells were cultured in alpha Modified Eagle's Medium (α-MEM, Gibco, Carlsbad, CA, USA) supplemented with 10% fetal bovine serum (FBS; Biological Industries, Kibbutz Beit Haemek, Israel), 1% L-glutamine (Sigma-Aldrich, St Louis, MO, USA), 1% penicillin and streptomycin (Amresco, Solon, OH, USA). Cells were maintained at a humidified 37°C, 5% CO_2_ incubator (Thermo Fisher Scientific, Waltham, MA, USA). All cell lines were tested for contamination.

### Establishment of MACF1 knockdown and overexpressed cell models

MACF1 overexpressed preosteoblast was constructed as described previously (Yin et al., 2018). Briefly, MC3T3-E1 cells (1×10^7^ per well) were electroplated (1800V and 30 ms) with the MACF1 overexpression plasmid pEGFP-C1A-ACF7 or control plasmid pEGFP-C1A using Neon Transfection System (Invitrogen) following the manufacturer's instructions. After the electroporation, cells were seeded into a six-well plate with 10 ml α-MEM and cultured for 6 h. Adherent cells were then washed with 2 ml α-MEM twice and the medium was changed to antibiotic-free growth medium (α-MEM with 10% FBS, and 1% L-glutamine). After being cultured for 48 h, the medium was changed to selective growth medium supplemented with 650 μg/ml Geneticin (MP Biomedicals, Santa Ana, CA, USA) and cells were continuously cultured for 2 weeks. MACF1 overexpression preosteoblast was monoclonalized by limited dilution method.

Stable MACF1-knockdown MC3T3-E1 cell line was established by transfection of lentivirus vector carrying shRNA targeting murine MACF1 (NM_001199136.1) or its scramble control as described previously (Hu et al., 2015, 2018).

### Migration assay *in vitro* and time-lapse videomicroscopy

For the wound healing assay, MC3T3-E1 cells with various MACF1 expression levels were cultured in growth medium to a confluence of 80–100% before the cell monolayer was scratched using a sterilized micropipette tip. After being washed twice with PBS, fresh growth medium was added. The images of the wounded area were captured after 0 h and 12 h after the scratch to monitor preosteoblast migration into the wounded area. The migratory abilities were quantified by measuring the distance between cells in the scratch zone.

For migration trajectory, the movement of individual preosteoblasts were traced by using time-lapse video microscopy. Cells were plated on fibronectin-coated dishes and imaged with an Olympus phase-contrast microscope (20×) for 6 h at three frames/h and manually. Transwell assay were performed using transwell inserted with a filter with an 8 μm pore size (BD Biosciences, San Jose, CA, USA). Preosteoblasts were detached and 2×10^4^ cells in serum-free medium were seeded into the upper chamber of the transwell insert. Culture medium with 10% FBS was added to the lower chamber. After 12 h incubation, cells that had migrated to the lower surface of inserts were stained with DAPI, photographed and counted from 10 randomly selected microscope fields.

### Dil labeling and cell implantation method

Preosteoblasts were seeded at a density of 2×10^5^ cells/well in six-well plates. After 48 h of culture, cells were detached with trypsin and washed with PBS twice. Then the cells were labeled with 5 μM of vibrant Dil cell labeling solution (Beyotime Biotechnology, China) for 30 min followed by washing twice with PBS. The Dil labeled (Dil^+^) cells were centrifuged and further cultured in standard conditions for 72 h until the pellet was formed.

For the calvarial defect model, 6-week-old BALB/c nude mice (male=4, female=4) were subjected to surgery to make a 1.5 mm diameter full thickness defect on the center of parietal bone using an electric cranial drill (JINKOU, USA). The Dil^+^ cell pellets were implanted into the defect. Then the skin wound on the calvaria was closed using 4-0 Nylon suture and mice were euthanized 2 weeks after surgery. Calvaria bones were fixed with 4% PFA and decalcified in 17% ethylene diamine tetraacetic acid (EDTA, Sigma-Aldrich, E9884) for 14 days, and embedded with OCT (optimal cutting temperature compound). Calvaria bones were dissected and visualized using a fluorescence microscope. Cell extension distance was measured using ImageJ software and statistically analyzed using the GraphPad Prism software. This study was performed in accordance with guidelines from the National Institutes of Health. All procedures performed on mice were approved by the Animal Care Committee of Northwestern Polytechnical University. BALB/c mice weighing 18–23 g, which were obtained from the laboratory animal center of the Xi'an Jiaotong University, were used for experiments.

### Immunocytochemistry and laser scanning confocal microscopy

Cells were seeded on FN-coated coverslips at a density of 1×10^4^/cm^2^ and cultured for 24 h. After 20 min fixation with 4% PFA and permeated with 0.5% Triton X-100 TBS, cells were blocked with 5% bovine serum albumin (BSA) in PBS for 30 min at room temperature. Then, cells were incubated with mouse α-tubulin antibody (1:50, no. 11224-1-AP, Proteintech, USA), rabbit EB1 antibody (1:50, no. 17717-1-AP, Proteintech, USA), rabbit GM130 antibody (1:50, no. 11308-1-AP, Proteintech, USA), rabbit p[Y418] Src antibody (1:50, no. ab4816, Abcam, USA) and mouse APC antibody (1:50, no. A2818, ABclonal, USA) in PBS containing 2% BSA overnight at 4°C. After washing with PBST (0.05% Tween 20), cells were incubated with Cy3-conjugated goat anti-rabbit (1:100, no. EK012, Zhuangzhi Biotechnology, Xi'an, China) and FITC conjugated goat anti-mouse IgG secondary antibody (1:100, no. EK011, Zhuang zhi Biotechnology, Xi'an, China) for 60 min at room temperature. 4′, 6-Diamidino-2-phenylindole (DAPI, 1 µg/ml) was used to counterstain cell nuclei for 10 min at room temperature. Each sample was washed, enveloped with Fluoromount-G (Southern Biotech, Birmingham, AL, USA) and examined with a laser scanning confocal microscope (Leica TCS SP5, Wetzlar, Germany). Cy3 was excited at a wavelength of 543 nm, FITC at 488 nm and DAPI at 405 nm and colocalization was analyzed by ImageJ software.

### Statistical analyses

All experiments were independently repeated at least three times with each done in triplicate. Statistical analyses of the data were performed using the GraphPad Prism 6 software (GraphPad Software, La Jolla, CA, USA), and a Student’s *t*-test was used. All data were reported as the mean±s.d., and *P*-values <0.05 were considered statistically significant for all comparisons.

## Supplementary Material

Supplementary information
