# Supplementary material for: MACF1 promotes preosteoblast migration by mediating focal adhesion turnover through EB1
Source: Biol Open. 2020 Mar 24;9(3):bio048173. doi: 10.1242/bio.048173 (PMC7104863; doi:10.1242/bio.048173)
Supplement: Supplementary information [file biolopen-9-048173-s1.pdf]

Supplementary data

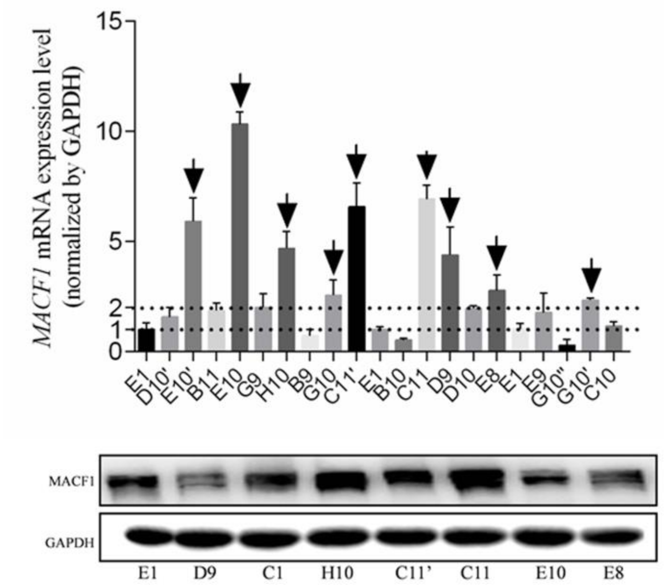

Figure. S1 The establishment of MACF1 overexpression MC3T3-E1 cell. MACF1 expression was detected by qPCR and western blot.

Biology Open • Supplementary information
